# Effect of low dose naloxone on the immune system function of a patient undergoing video-assisted thoracoscopic resection of lung cancer with sufentanil controlled analgesia — a randomized controlled trial

**DOI:** 10.1186/s12871-019-0912-6

**Published:** 2019-12-19

**Authors:** Yun Lin, Zhuang Miao, Yue Wu, Fang-fang Ge, Qing-ping Wen

**Affiliations:** 1 Department of Anesthesiology, The First Affiliated Hospital of Dalian Medical University, No.193 Lian he Road, Xi gang District, Dalian City, Liaoning Province 116000 People’s Republic of China; 2 Dalian Medical of University, Dalian, China

**Keywords:** Low-dose naloxone, Opioid growth factor, Immune function, Postoperative pain, Nausea, Vomiting

## Abstract

**Background:**

Perioperative immune function plays an important role in the prognosis of patients. Several studies have indicated that low-dose opioid receptor blockers can improve immune function.

**Methods:**

Sixty-nine patients undergoing video-assisted thoracoscopic resection of the lung cancer were randomly assigned to either the naloxone group (*n* = 35) or the non-naloxone group (*n* = 34) for postoperative analgesia during the first 48 h after the operation. Both groups received sufentanil and palonosetron via postoperative analgesia pump, while 0.05 μg·kg^− 1^·h^− 1^ naloxone was added in naloxone group. The primary outcomes were the level of opioid growth factor (OGF) and immune function assessed by natural killer cells and CD4^+^/CD8^+^ T-cell ratio. Second outcomes were assessed by the intensity of postoperative pain, postoperative rescue analgesia dose, postoperative nausea and vomiting (PONV).

**Results:**

The level of OGF in the naloxone group increased significantly at 24 h (*p*<0.001) and 48 h after the operation (*P* < 0.01). The natural killer cells (*P* < 0.05) and CD4^+^/CD8^+^ T-cell ratio (*P* < 0.01) in the naloxone group increased significantly at 48 h after the operation. The rest VAS scores were better with naloxone at 12 and 24 h after operation(*P* < 0.05), and the coughing VAS scores were better with naloxone at 48 h after the operation(*P* < 0.05). The consumption of postoperative rescue analgesics in the naloxone group was lower (0.00(0.00–0.00) vs 25.00(0.00–62.50)), *P* < 0.05). Postoperative nausea scores at 24 h after operation decreased in naloxone group(0.00 (0.00–0.00) vs 1.00 (0.00–2.00), *P* < 0.01).

**Conclusion:**

Infusion of 0.05 μg·kg^− 1^·h^− 1^ naloxone for patients undergoing sufentanil-controlled analgesia for postoperative pain can significantly increase the level of OGF, natural killer cells, and CD4+/CD8+ T-cell ratio compared with non-naloxone group, and postoperative pain intensity, request for rescue analgesics, and opioid-related side effects can also be reduced.

**Trial registration:**

The trial was registered at the Chinese Clinical Trial Registry on January 26, 2019 (ChiCTR1900021043).

## Background

Cancer has become a major public health concern all over the world, among which lung cancer is a prominent problem. Surgical resection is the principal treatment for tumors [1, 2]. Recurrence and metastasis of tumors are the main causes of death in patients with lung cancer [3]. The perioperative periods are a dangerous time points for tumor recurrence and metastasis. Immunosuppression plays a significantly important role in the development of tumors [4]. Improvement of postoperative immune function is vitally important for patients. In addition, appropriate postoperative pain control, and effective management of postoperative nausea and vomiting (PONV) lead to several benefits, including earlier restoration of mobility, shorter hospital stays, lower hospital costs and higher comfort and satisfaction of patients.

Opioid receptor antagonists such as naloxone are widely used in the clinical setting to treat opioid-induced respiratory depression and drug addiction. Regulation of endogenous opioids by opioid receptor antagonists may explain the role of opioid peptide-opioid receptor interactions in many biological processes and diseases [5]. One of the functions of endogenous opioids is the regulation of cell growth [6]. Studies have shown that one of the endogenous opioids called opioid growth factor (OGF, chemically termed [MET^5^]-Enkephalins) enhances the immune function by increasing the number of natural killer cells (NK cells), T-cells and the levels of interleukin-2 [7–9].

Gans et al. were among the first to report that morphine requirement was significantly less in patients receiving low-dose naloxone, and the finding suggested that low-dose naloxone enhanced analgesia [10]. Moreover, several studies have shown that low-dose naloxone might enhance analgesia and reduce opioid-related adverse effects, such as nausea and vomiting and pruritus [11–13]. Studies showed that low-dose naloxone may enhance analgesic effects through increasing the release of endogenous opioids and up-regulating opioid receptor [14–16]. Some studies further suggested that low-dose naloxone may improve the analgesic effects by releasing enkephalin [13]. However, a survey of the literature shows that little is known about the effects of low dose naloxone on the immune system function of a patient undergoing video-assisted thoracoscopic resection of lung cancer with sufentanil-controlled analgesia. This study aimed to explore the effects of low dose infusion of naloxone 0.05 μg·kg^− 1^·h^− 1^ on a patient undergoing video-assisted thoracoscopic resection of lung cancer with sufentanil-controlled analgesia.

## Methods

This randomized controlled trial was reported according to the Consolidated Standards of Reporting Trials (CONSORT) guidelines and conducted after the approval of the Ethics Committee of the First Affiliated Hospital of the Dalian Medical University on January 24, 2019 (protocol number: PJ-KY-2018-141(X)). Written informed consent was obtained from patients after providing them with adequate explanation regarding the aims of this study. The trial was registered at the Chinese Clinical Trial Registry before patients’ enrolment (www.chictr.org.cn, number: ChiCTR1900021043) on January 26, 2019, with Lin Yun as principal investigator. The trial completed a pilot study of 20 patients to calculate the sample size of this trial. The pilot study was performed from February 1, 2019 to February 16, 2019, and the patient data were included in this trial. We enrolled 70 patients aged 18 to 65 with American Society of Anesthesiology physical status II to III undergoing video-assisted thoracoscopic resection of the lung cancer. Patients with severe cardiopulmonary, liver or kidney diseases, allergy to naloxone, opioid addiction or drug abuse, and vertigo were excluded.

Upon arrival in the operation room, standard monitoring was determined. Anesthesia was induced with midazolam, sufentanil, cisatracurium, and propofol, subsequently intubation with double lumen tube and location by fiber bronchoscope. Ventilator parameters were adjusted to maintain pulse saturation of oxygen (SpO_2_) 95–100% and end-tidal carbon dioxide between 35 and 40 mmHg. Anesthesia was maintained with propofol, remifentanil and cisatracurium and the depth of anesthesia was maintained at a bispectral index value of 40 to 60. The postoperative analgesic pump was used at the end of the operation. Sufentanil 0.04 μg·kg^− 1^·h^− 1^ (calculated at 48 h), palonosetron 0.5 mg and saline diluted to 100 mL were used in a non-naloxone group, while 0.05 μg·kg^− 1^·h^− 1^ naloxone (calculated at 48 h) was added in naloxone group. PCA was set to administer a bolus dose of 2 mL with a lockout interval of 20 min and a background infusion rate of 2 mL/h. Patients were randomly allocated into 2 groups (1:1 allocation ratio) by a sequence generated from a pseudorandom number seed. Because other non-opioid drugs may have different effects on immune function, postoperative rescue analgesia was chosen to perform intramuscular injection with meperidine in both groups. All patients in both groups were instructed on how to use the PCA device and on how to use the visual analogue scale (VAS) to rate the intensity of the pain at rest or while coughing and nausea on a scale from 0 to 10 (with 0 denoting the lowest level of intensity of the symptom and 10, the worst imaginable intensity).

The primary outcomes of the study were the levels of OGF and postoperative immune function assessed by NK cells and CD4^+^/CD8^+^ T-cell ratio. Second outcomes were assessed by the VAS scores of postoperative pain, nausea and analgesic dose, inflammatory responses measured by white blood cell (WBC) count and neutrophil percentage, respiratory depression, and hospital stay. Immune function and inflammatory responses were measured before the surgery, at 24 and 48 h after surgery. Both groups of patients rated the intensity of their pain with VAS and respiratory depression 1, 6, 12, 24 and 48 h after the operation (respiratory depression: respiratory rate ≤ 8/min or SpO_2_<90%). Both groups of patients rated the scale of nausea and the dose of Meperidine at 24 and 48 h after operation and hospital stay.

### T lymphocyte subsets and natural killer cells assay

Venous blood samples were taken before the surgery, and 24 and 48 h after surgery. Moreover, flow cytometry (BD Company, USA) was applied to assess the changes in peripheral blood T lymphocyte subsets (CD3^+^, CD4^+^, CD8^+^, and CD4^+^/CD8^+^ T-cell ratio) and NK cells.

### OGF assay

Venous blood samples were taken before the surgery, 24 and 48 h after surgery. OGF was measured in serum using a commercial ELISA kit (MEK (Methionine-Enkephalin) ELISA kit; Elabscience.).

### Statistical analysis

The primary aims of this study were to determine the differences in the levels of OGF, NK cells and CD4^+^/CD8^+^ T-cell ratio and the secondary outcomes including VAS scores of postoperative pain, nausea, postoperative rescue analgesia dose, WBC count, neutrophil percentage, respiratory depression and hospital stay in naloxone and control groups. Results were expressed as means ± SD, medians with interquartile range, or numbers and percentages of participants as appropriate. The demographics and intraoperative situations were compared by Student *t* test or χ^2^ test. Fisher’s exact test was used for small sample sizes (expected frequencies < 5). The levels of OGF, NK cells, CD4^+^/CD8^+^ T-cell ratio, WBC count, neutrophil percentage and hospital stay analyzed with a one-way ANOVA between the two groups, and non-normally distributed variables were analyzed with the Mann-Whitney U test. *P* values < 0.05 were considered significant. Statistical analysis was performed using SPSS version 22.0.

A pilot study was performed prior to patient recruitment to estimate an appropriate sample size. The pilot study included 20 subjects, 10 in each arm. We calculated the primary outcome of the study assessed by NK cells. The sample size of 32 participants each group provided α = 0.05, 80% power, and an allocation ratio = 1.0. Accounting for loss of data, each group needed 35 patients. The sample calculation was performed with PASS version 11.0.

## Results

**Fig. 1:**
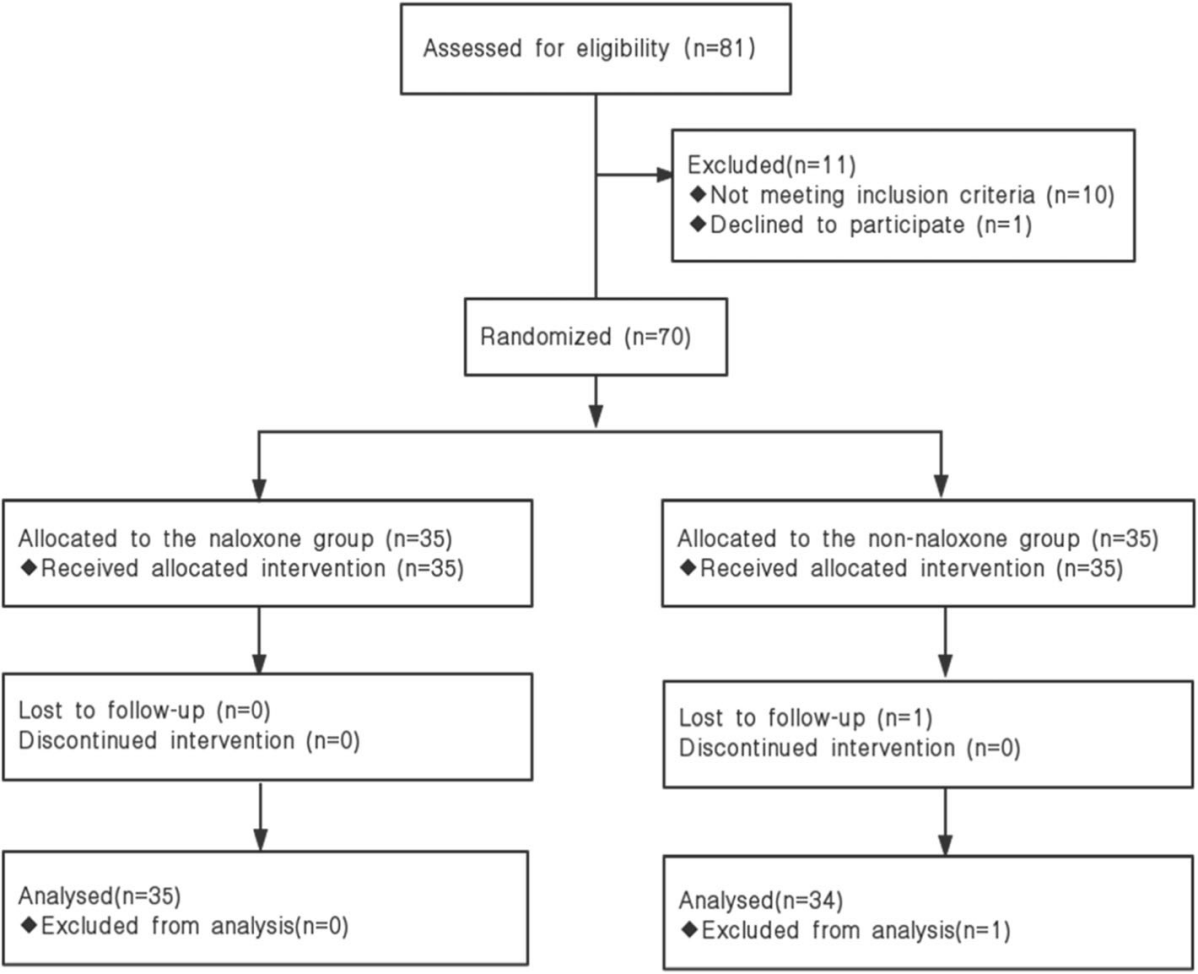
Diagram Showing Flow of Study Participants

Among the 81 patients assessed for eligibility, 70 patients were enrolled and randomly assigned to the groups, and 69 patients completed the study (Fig. 1). Data from one patient was excluded from the analysis due to early discharge (the next day after the operation). There were no significant differences in patient characteristics (Table 1).

**Table 1:** Patient characteristics

	Naloxone(*n* = 35)	Non-naloxone (*n* = 34)	*P* Value
Age(y)	55.46 ± 8.65	55.46 ± 8.65	0.378
Gender, N(%)			0.900
Male	17(48.57)	16(47.06)	
Female	18(51.43)	18(52.94)	
Body mass index (kg/m^2^)	25.42 ± 4.19	24.52 ± 3.67	0.351
ASA physical status, N (%)			1.000
II	31(88.57)	30(11.76)	
III	4(11.43)	4(88.24)	
Type of operation, N (%)			0.777
Video-assisted thoracoscopic pulmonary lobectomy	25(71.43)	26(76.47)	
Video-assisted thoracoscopic pulmonary wedge resection	9(25.71)	8(23.93)	
Video-assisted thoracoscopic pulmonary segmentary	1(2.86)	0(0.00)	
Duration of operation (min)	150.00 (120.00–180.00)	180.00 (120.00–180.00)	0.339
Fluid intake (mL)	1000.00(1000.00–1000.00)	1000.00(1000.00–1000.00)	0.854
Blood loss (mL)	113.71 ± 38.278	106.76 ± 36.987	0.446
Type of cancer, N (%)			0.780
Carcinoma in situ	8(22.85)	6(17.65)	
Microinvasive adenocarcinoma	14(40.00)	11(32.35)	
infiltrating adenocarcinoma	12(34.29)	14(41.18)	
Mucinous adenocarcinoma	1(2.86)	2(5.88)	
Moderately differentiated adenocarcinoma	0(0.00)	1(2.94)	
Lymphatic metastasis			0.614
Yes	1(2.86)	2(5.88)	
No	34(97.14)	32(94.12)	

*ASA* American Society of Anesthesiologists

**Fig. 2:**
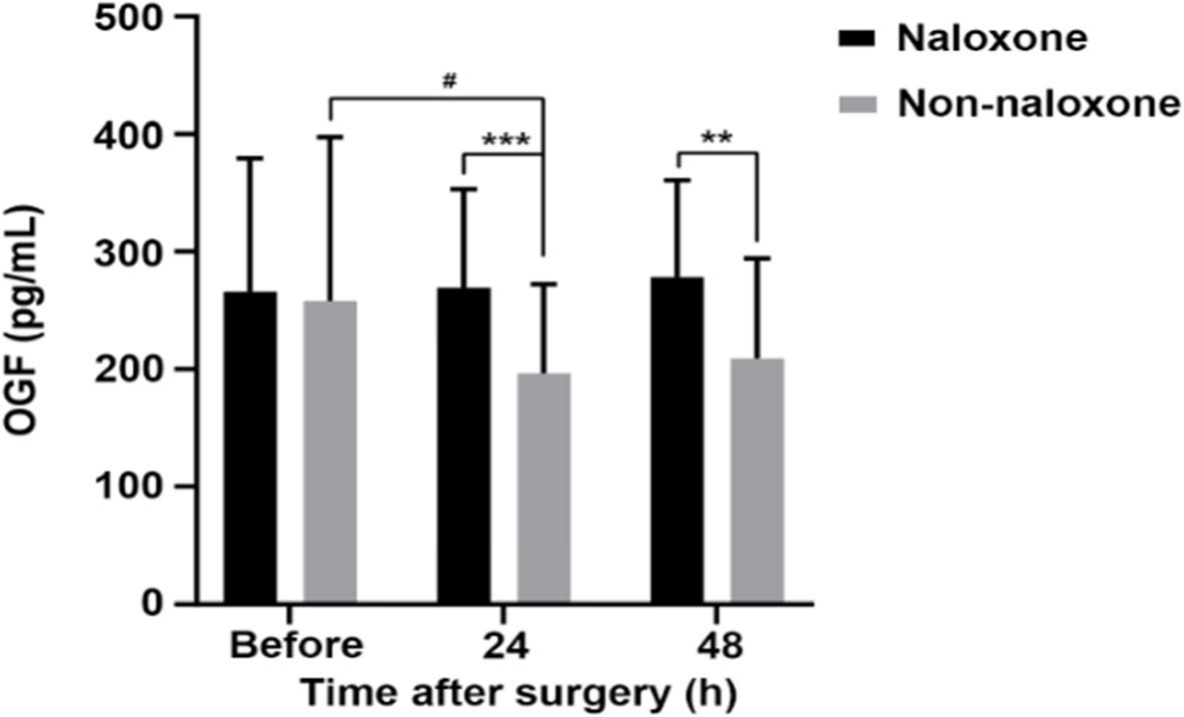
Changes in OGF levels after surgery. OGF levels are presented as means ± SD. Significantly different from the non-naloxone group at ^**^P<0.01, ^***^P<0.001. ^#^*P*<0.05 versus “before surgery” for each group

**Fig. 3:**
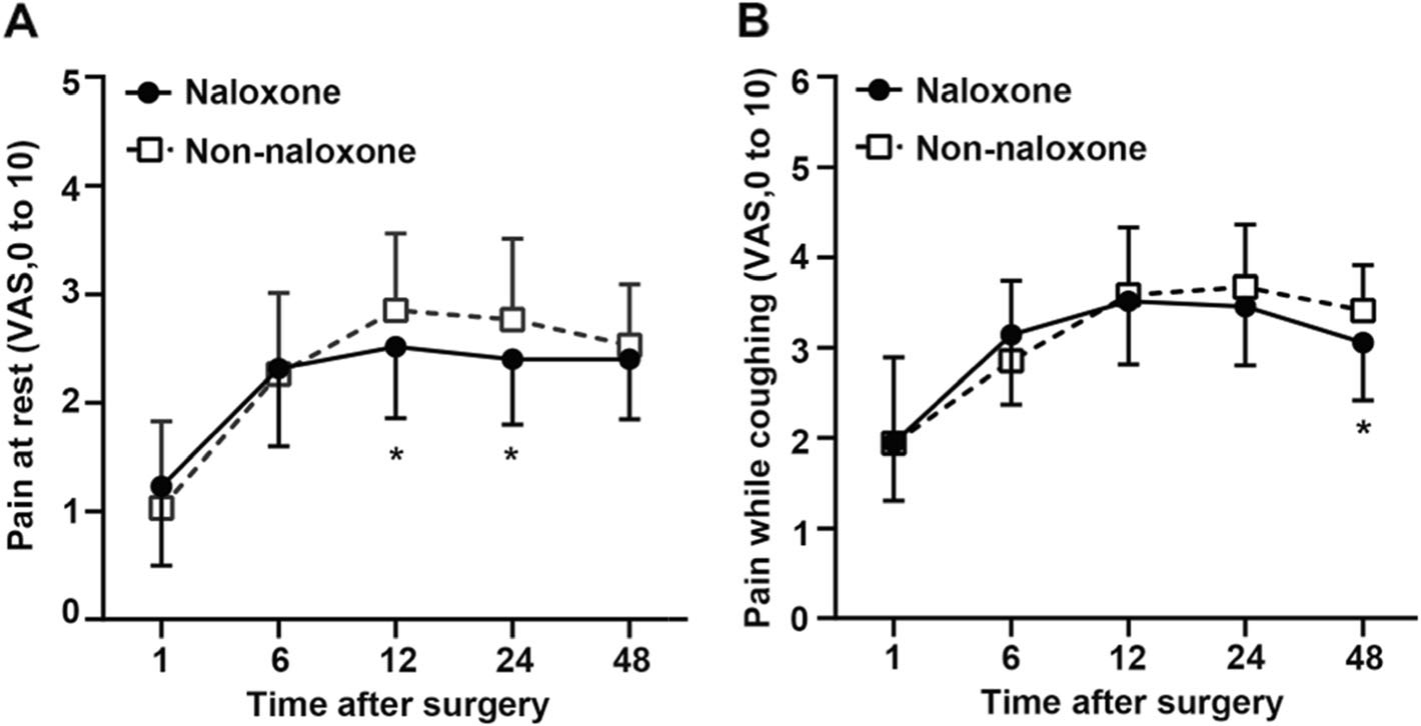
Visual analog scale for pain (**a**) at rest and (**b**) while coughing 1, 6, 12, 24, and 48 h after surgery. Data are expressed as means ± SD. Significantly different from the non-naloxone group at ^*^P<0.05

The levels of OGF in the naloxone group were significantly higher at 24 h(*p*<0.001) and 48 h after the operation (*P* < 0.01) in Fig. 2. NK cells (*P* < 0.05) (Table 2) and CD4^+^/CD8^+^ T-cell ratio (*P* < 0.01) (Table 3) in patients from the naloxone group significantly increased compared with non-naloxone group at 48 h after the operation. There were no significant differences in the NK cells (Table 2) and CD4^+^/CD8^+^ T-cell ratio (Table 3) at 24 h after the operation. The rest VAS scores were better with naloxone at 12 and 24 h after the operation(*P* < 0.05) (Fig. 3). The coughing VAS scores were also better with naloxone at 48 h after the operation (*P* < 0.05) (Fig. 3). There were no significant differences at other time points in Fig. 3. The rescue postoperative analgesics dose injected in patients from the naloxone group was 0.00(0.00–0.00) mg lower compared with 25.00(0.00–62.50) mg injected in patients from the non-naloxone group (*P* < 0.05) (Table 4).

**Table 2:** Changes in NK Cells After Surgery

		Naloxone(*n* = 35)	Non-naloxone (*n* = 34)	*P* Value
NK cells(%)	Before surgery	16.14 ± 5.75	16.63 ± 6.40	0.519
	24 h after operation	14.13 ± 6.28	14.53 ± 5.85	0.918
	48 h after operation	15.97 ± 5.44	13.06 ± 5.47^#^	0.030

*NK cells* Natural Killer Cells. ^#^*P*<0.05 versus “before surgery” for each group

**Table 3:** Changes in T cells After Surgery

		Naloxone(*n* = 35)	Non-naloxone(*n* = 34)	*P* Value
CD3^+^T cells(%)	Before surgery	56.94 ± 9.01	56.95 ± 8.98	0.997
	24 h after operation	46.22 ± 12.67^###^	41.48 ± 9.99^###^	0.089
	48 h after operation	56.17 ± 8.96	53.36 ± 10.58	0.237
CD4^+^T cells(%)	Before surgery	33.49 ± 6.92	32.61 ± 5.52	0.560
	24 h after operation	25.39 ± 8.55^###^	21.20 ± 7.81^###^	0.037
	48 h after operation	32.70 ± 6.39	28.91 ± 6.11^#^	0.014
CD8^+^T cells(%)	Before surgery	23.45 ± 4.37	24.34 ± 5.09	0.437
	24 h after operation	20.83 ± 7.02^#^	20.28 ± 6.54^##^	0.734
	48 h after operation	23.47 ± 4.10	24.57 ± 6.21	0.391
CD4^+^/CD8^+^ T cell ratio	Before surgery	1.49 ± 0.36	1.39 ± 0.30	0.226
	24 h after operation	1.32 ± 0.51	1.15 ± 0.52^#^	0.163
	48 h after operation	1.41 ± 0.27	1.21 ± 0.29	0.003

*CD* Clusters of Differentiation

^#^*P*<0.05 versus “before surgery” for each group. ^##^*P*<0.01 versus “before surgery” for each group, ^###^*P*<0.001 versus “before surgery” for each group

**Table 4:** Rescue Analgesic Dose, Postoperative Nausea and Vomiting scores, Respiratory Depression and Hospital Stay

	Naloxone(*n* = 35)	Non-naloxone (*n* = 34)	*P* Value
Rescue analgesic dose (mg)	0.00(0.00–0.00)	25.0(0.00–62.50)	0.034
Nausea VAS score 24 h after operation	0.00(0.00–0.00)	1.00(0.00–2.00)	0.001
Vomiting 24 h after operation, n(%)	6(17.14)	11(32.35)	0.143
Nausea VAS score 48 h after operation	0.00(0.00–0.00)	0.00(0.00–0.00)	0.318
Vomiting 48 h after operation, n(%)	3(8.57)	5(14.71)	0.675
Respiratory depression 1 h after operation, n(%)	0.00(0.00)	0.00(0.00)	>0.99
Respiratory depression 6 h after operation, n(%)	0.00(0.00)	0.00(0.00)	>0.99
Respiratory depression 12 h after operation, n(%)	0.00(0.00)	0.00(0.00)	>0.99
Respiratory depression 24 h after operation, n(%)	0.00(0.00)	0.00(0.00)	>0.99
Respiratory depression 48 h after operation, n(%)	0.00(0.00)	0.00(0.00)	>0.99
Hospital stay (day)	4.66 ± 1.39	5.35 ± 1.77	0.074

Table 4 showed that postoperative nausea scores significantly decreased in patients from the naloxone group(0.00 (0.00–0.00) vs. 1.00(0.00–2.00), *P* < 0.01) at 24 h after the operation. There were no significant differences in nausea scores between the groups at other time points (Table 4). There were no significant differences in the postoperative vomiting after the operation (Table 4). And there were no significant differences in the postoperative hospital stay (P>0.05) in Table 4. The data showed no significant differences in the postoperative inflammatory responses assessed by WBC count and the percentage of neutrophil between the two groups (*P*>0.05) in Table 5.

**Table 5:** Changes in White Blood Cell Count and Neutrophil Percentage After Surgery

		Naloxone(*n* = 35)	Non-naloxone (*n* = 34)	*P* Value
WBC count/uL	Before surgery	7.81 ± 1.23	7.78 ± 1.25	0.907
	24 h after operation	11.96 ± 3.13^###^	13.21 ± 3.41^###^	0.115
	48 h after operation	9.93 ± 2.86^##^	10.79 ± 2.87^###^	0.214
Neutrophil Percentage(%)	Before surgery	71.74 ± 7.06	72.49 ± 6.71	0.656
	24 h after operation	83.57 ± 5.71^###^	85.48 ± 4.39^###^	0.124
	48 h after operation	76.47 ± 8.55^##^	79.41 ± 6.67^###^	0.117

^##^*P*<0.01 versus “before surgery” for each group. ^###^*P*<0.001 versus “before surgery” for each group

## Discussion

In this study, we found that 0.05 μg·kg^− 1^·h^− 1^ naloxone for patients with sufentanil-controlled analgesia could increase the levels of OGF, NK cells and CD4^+^/CD8^+^ T-cell ratio compared with non-naloxone group. Studies have shown that OGF could increase the number of NK cells and T cells [7, 8]. NK cells have been showed to play an important role in effective immune responses and immunosurveillance. Cytolytic enzymes and cytokines produced by NK cells, like IFN-γ, are beneficial to inhibit cancer cells [17]. The survival of lung cancer patients was closely related to the level of NK cells [18], and patients’ survival rates are bound up with the level of NK cells in primary squamous cell lung carcinoma [19]. The decline of NK cells leads to the occurrence and development of tumors [20, 21] and the level of the NK cells is of great significance in judging clinical prognosis. Our results showed that low-dose naloxone may inhibit tumors by increasing the level of NK cells regulated by OGF.

T cell subsets play a major role in cellular immunity. The number of CD3^+^ T cells represents the overall cellular immune status of the body. CD8^+^ is a cytotoxic T lymphocyte, which can eradicate virally infected cells and cancer cells, trigger apoptosis by release of cytotoxins and directly contact with cells [22]. CD4^+^ is a T helper cell with the function of immune regulation, which can recognize the antigens produced by tumor and inhibit cancer cells by activating other immune cells, such as NK cells and cytotoxic T lymphocyte, and NK cells also play a role in activation of cytotoxic T cell [23]. In addition, cytokines secreted by NK cells have effect on T helper cell polarization [17]. The maintenance of normal immune function depends on the cooperation or restriction between various immune cells (especially all kinds of T cell subsets and NK cells). CD4^+^/CD8^+^ T-cell ratio reflect the immune status of the body [24]. In physiological state, CD4^+^/CD8^+^ T-cell ratio is relatively constant. The decrease of CD4^+^/CD8^+^ T-cell ratio indicates the decrease of immune function and the severity of disease or poor prognosis [24]. The results showed that CD4^+^/CD8^+^ T-cell ratio in naloxone group was higher 48 h after the operation, suggesting that low dose naloxone may enhance cellular immunity and anti-tumor effects. Low-dose naloxone may enhance immune function by decreasing pain intensity, but whether the increase of CD4^+^/CD8^+^ T-cell ratio is related to OGF is uncertain.

The postoperative immune function may be related to operation, anesthesia, postoperative pain, body temperature and blood transfusion in the operation, etc [4, 25–27]. Some studies indicated that the operation itself and stress responses induced by operation could result in a reduction of the postoperative NK cells [28]. The effects of anesthesia on immune function have been widely discussed in recent years, but the result is still controversial. Opioid drugs may cause postoperative immunosuppression by reducing the number of NK cells [29, 30], but it is difficult to control the stress and pain caused by surgical stimulation without the use of opioids, and the acute pain could activate the hypothalamus-pituitary-adrenal (HPA) axis, which in turn has an effect on the number of NK cells [31]. And there are other non-opioid drugs that affect immune function [29]. The combination of these factors may result in our results that number of NK cells in the naloxone group was higher than that of non-naloxone group after operation, but the number of NK cells in the both of two groups were lower compared to the time before surgery.

Many experiments have been carried out to evaluate the effects of low-dose naloxone on postoperative analgesia and opioid-related side effects. The analgesic effects and adverse effects of opioids are dose-dependent. The dose of naloxone administration in the report provided highly variable ranging from 0.008 μg·kg^− 1^·h^− 1^ to 0.57 μg·kg^− 1^·h^-1^ [32]. The reason why 0.05 μg·kg^− 1^·h^− 1^ naloxone was chosen in our study was that patient-controlled intravenous analgesia (PCIA) with this dose in YAO’s experiment confirmed that low-dose naloxone increased the analgesic effects by increasing the levels of endogenous opioid peptides [16]. The data showed that the rest pain scores decreased significantly at 12 and 24 h after surgery and coughing pain scores decreased significantly at 48 h after surgery, and the rescue analgesic dose after surgery was lower in the naloxone group, indicating that low-dose naloxone could enhance the analgesic effects of sufentanil and reduce the dose of analgesic. Data showed that the coughing VAS scores were at a higher level than that of rest VAS scores after operation, and the patients in the two groups all showed low tolerance while coughing. It may be the reason for patients not to feel obviously relief at higher level of pain due to the subjectivity of VAS scores. This may explain why the difference happened at 48 h after surgery for the pain on coughing, but for the pain at rest, the difference happened at 12 and 24 h after surgery.

The VAS scores of postoperative nausea decreased significantly on the first day after the operation. The mechanism of the effects of low-dose naloxone on analgesic efficacy and opioid-related side effects is not clear. In addition to releasing enkephalins [33], it is believed that the functions of the μ-opioid receptor excitatory G-protein complexes (GS) are antagonized by naloxone at a low dose, triggering improvement of analgesic effects and reduction in adverse effects such as nausea and vomiting [13]. Some studies also indicated that low dose of naloxone could reduce neuropathic pain by lowering the levels of inflammatory factors [34]. Our study found that the levels of OGF increased significantly two days after the operation, suggesting that the mechanism of low-dose naloxone enhancing the analgesic effects of sufentanil, reducing opioids consumption and postoperative nausea may be related to the level of endogenous OGF.

We have always attached great importance to postoperative analgesia management. Our results showed that postoperative pain in rest can be well controlled, but the pain scores on coughing were overall on the high level. Patients were encouraged to mobilize out of bed early and cough after the thoracoscopic surgery in order to reduce postoperative complications. This promote us to control the VAS scores at a lower level during coughing and activities to ensure patient’s favorable prognosis and satisfaction. At the same time, we also found that opioids had effective analgesia on coughing but the frequency of postoperative nausea and vomiting after operation was higher. Hence, with respect to the better postoperative management, we would like to find a way to control the occurrence of postoperative nausea and vomiting in patients, not just the postoperative acute pain. And we hope to find a good balance between the control of postoperative nausea and pain. Low-dose naloxone may be a good choice from the experimental results, but more experiments are needed to prove this possibility.

We noticed the basic studies have shown that the regimen of short-term exposure to naltrexone appeared to lead to enhanced interaction of the up-regulated OGF [33]. Blockade of opioid peptides from opioid receptors for a short period each day (4–6 h), using a daily administration of low-dose naltrexone (LDH), provides an 18-20 h window wherein the elevated levels of endogenous opioids and opioid receptors can interact to elicit a response [33]. However, in this study, we used 0.05 μg·kg^− 1^·h^− 1^ naloxone continuous infusion along with sufentanil PCIA for about 48 h, and the levels of OGF increased significantly within 48 h. There may be two possibilities for this difference, one of which may be related to the difference of half-time of naloxone and naltrexone. Both naltrexone and naloxone are opioid receptor antagonists and have no intrinsic activity, but the duration of naltrexone blockade is about 3–4 times longer than that of naloxone. The other one may be associated with different dose. According to the potency relationship between naltrexone and naloxone, low dose naloxone (4.5 mg) [35] in the report was far more than 0.05 μg·kg^− 1^·h^− 1^ naloxone we used in this study. Although we used continuous naloxone infusion for 48 h, the shorter blockade duration and lower dose might not block all opioid receptors. These may be the reason why the elevated levels of OGF can still interact with its receptor to elicit a response. The mechanism of the increase in the levels of OGF is that low-dose naloxone may cause excessive release of endogenous opioids through blockade of presynaptic auto-inhibition of enkephalin release [13].

There are two limitations in this study. First, the duration of immune function detected was limited to 48 h after the operation, and we did not further observe changes of immune indexes. The experimental data showed that there were no significant changes in the immune function on the first day after the operation. The NK cells and CD4^+^/CD8^+^ T-cell ratio in naloxone group began to be higher on the second day after the operation. If we continue to test NK cells and T cells, we could explore the extent and duration of using low-dose naloxone to improve immune function with PCA after the operation. Second, the long-term prognosis of the patients was not observed. Studies have shown that OGF can not only enhance immune function but also directly inhibit tumors. OGF activates the Rb pathway by up-regulating p16 and/p21, which are cyclin-dependent inhibitory kinases, with delayed cell replication and ultimate cell number resulting [36]. Thus, low-dose opioid receptor antagonists mediated modulation of the OGF-OGFr axis appears to account for the depressed DNA synthesis and proliferation of cancer cells [33]. OGF may inhibit the recurrence and metastasis of tumors after surgery. Since no further follow-up of the patients’ OGF levels and recurrence or metastasis after surgery between the groups, it was not observed that whether low-dose naloxone could directly affect the occurrence and development of tumors through OGF-OGFr.

## Conclusion

In conclusion, 0.05 μg·kg^− 1^·h^− 1^ naloxone increased the number of NK cells, CD4^+^/CD8^+^ T cell ratio and the analgesic effects after thoracoscopic resection of lung cancer on PCIA, meanwhile reduced analgesics dose and PONV after the operation. The enhancement of immune function and the analgesic effects of sufentanil and reduction of PONV may be related to the increased level of endogenous OGF.

## Data Availability

The datasets used or analyzed during the current study are available from the corresponding author on reasonable request.

## Abbreviations

ASA: American Society of Anesthesiologists
CD: Clusters of Differentiation
GS: G-protein complexes
LDH: Low-dose naltrexone
NK cells: Natural killer cells
OGF: Opioid growth factor
PCA: Patient controlled analgesia
PCIA: Patient-controlled intravenous analgesia
PONV: Postoperative nausea, and vomiting
SpO_2_: Pulse saturation of oxygen
T cells: T lymphocytes
VAS: Visual analogue scale
WBC: White blood cell
